# GlomSAM: Hybrid customized SAM for multi-glomerular detection and segmentation in immunofluorescence images

**DOI:** 10.1371/journal.pone.0321096

**Published:** 2025-04-14

**Authors:** Shengyu Pan, Xuanli Tang, Bingxian Chen, Xiaobo Lai, Wei Jin

**Affiliations:** 1 School of Medical Technology and Information Engineering, Zhejiang Chinese Medical University, Zhejiang, Hangzhou, China; 2 Department of Nephrology, Hangzhou TCM Hospital Affiliated to Zhejiang Chinese Medical University, Hangzhou, China; 3 Ningbo KonFoong Bioinformation Tech Co. Ltd, Ningbo, Zhejiang, China; South China University of Technology, CHINA

## Abstract

In nephrology research, multi-glomerular segmentation in immunofluorescence images plays a crucial role in the early detection and diagnosis of chronic kidney disease. However, obtaining accurate segmentations often requires labor-intensive annotations and existing methods are hampered by low recall rates and limited accuracy. Recently, a general Segment Anything Model (SAM) has demonstrated promising performance in several segmentation tasks. In this paper, a SAM-based multi-glomerular segmentation model (GlomSAM) is introduced to employ SAM in the immunofluorescence pathology domain. The fusion encoder strategy utilizing the advantages of both convolution networks and transformer structures with prompts is conducted to facilitate SAM’s transfer learning in applications of pathological analysis. Moreover, a rough mask generator is employed to generate preliminary glomerular segmentation masks, enabling automated input prompting and improving the final segmentation results. Extensive comparative experiments and ablation studies show its state-of-the-art performance surpassing other relevant research. We hope this report will provide insights to advance the field of glomerular segmentation and promote more interesting work in the future.

## Introduction

Chronic kidney disease (CKD) has gained worldwide recognition as a major health issue, posing significant socioeconomic challenges [1]. As CKD progresses from early to advanced stages, patients often require costly renal replacement therapies like dialysis or kidney transplantation to survive. Early detection and intervention are therefore critical to mitigate the progression of CKD and reduce its associated burdens. Renal biopsy and pathology have been used for the diagnosis and therapeutic management of both acute and chronic kidney diseases for a long time [2]. Clinically, renal biopsy specimens are usually evaluated by light microscopy, electron microscopy, and immunofluorescence (IF) microscopy in combination [3]. Moreover, IF staining has become the gold standard for diagnosing some kinds of diseases such as IgA nephropathy (IgAN) and Membranous nephropathy (MN) which are the first and second most common CKD in China [4].

The glomerular lesion is the key information reflecting the etiology of CKD in IF images, therefore recognizing pathological manifestations is crucial [5]. It is necessary to use several profiles to reflect richer information about the lesions since lesions can appear at any location. The diagnosing steps for renal pathologists always include glomerular finding and detail analyzing [6]. This requires diagnosis and fusion of information from all the glomeruli at multiple indicators (including IgG, IgM, IgA, kappa, and lambda light chains). For instance, diseased glomeruli are characterized by the deposition of immunocomplexes in the medical diagnosis of IgAN, predominantly containing IgA [7]. This deposition leads to mesangial cell proliferation, matrix expansion, and accumulation of complement C3 along with other membrane attacks complexes in the glomerular mesangial region [8]. Therefore, scanning these glomeruli from whole slide images (WSI) of multiple indicators is a time-consuming and labor-intensive process [9].

In recent years, deep learning and machine learning methods have proven to be very effective in advancing the field of digital pathology domain, which focuses on diagnosing and quantifying diseases by analyzing medical images acquired from scanned pathological tissue samples [10]. Technically, pathology image analysis includes classification, segmentation, detection, and assisted diagnosis. Much researches have focused on WSI from H&E, PAS, Masson trichrome, or some other materials, and obtained high-performance results [11–13]. However, there are few studies of identifying and segmenting glomeruli on IF images of renal biopsies, which is a fundamental and crucial step in the automatic pipeline. It is hard to identify glomeruli on IF images because of the unclear boundary and similar color just like some camouflage situations. For example, Peng et al. located glomeruli on several common detectors such as Faster R-CNN, RFCN, Mask R-CNN, and SSD in IF-WSI, obtaining bounding boxes without detailed boundaries [14]. Liu et al. selected UNet++ for preprocessing in the proposed pipeline and the Dice similarity coefficient (DICE) for the original image segmentation was 66.35% [15]. Govind et al. applied a butterworth bandpass filter to extract glomeruli and got a DICE of 83% [16]. So, there is still a large improvement room.

Existing methods lack sufficient feature representation and extraction capabilities due to model size limitations, making it challenging to capture glomerular morphology and characteristics in IF images comprehensively. Moreover, these models often require large amounts of annotated data for training, while obtaining labeled glomerular samples is costly as it requires professional pathologists for annotation.

Recently, Segment Anything Model (SAM) [17] and SAM2 [18] have pushed the boundaries of segmentation, demonstrating significant generalization and zero-shot inference capabilities. Using large dataset training to obtain a pretraining model grants SAM computational efficiency, and robust ability, which is crucial for large-scale kidney WSI applications that involve processing vast amounts of data [19]. Variants of the SAM have demonstrated superior performance in diverse image segmentation domains, attracting much attention in the academic community. For example, its medical domain variant MedSAM demonstrates distinct advantages in processing images with complex backgrounds and fine details. To address the aforementioned challenges, including model size limitations and the scarcity of annotated data, this paper proposes Glomerular-SAM (GlomSAM) based on SAM to better improve the detection and segmentation performance on IF images. In summary, the main contributions are as follows:

- A novel instance segmentation algorithm based on SAM for glomerular immunofluorescence images named GlomSAM is proposed. High-quality transfer of SAM into the kidney pathology field for glomerular identification task is achieved through hybrid customized strategies with parameter efficient fine-tuning technology.
- A CNN branch is introduced into the image encoder to enhance the model’s ability to learn the edges, textures, and local patterns of glomeruli, by utilizing cross-branch attention to fuse CNN’s advantage in capturing local features. Meanwhile, a Prompt Generator is incorporated into the Vision Transformer (ViT) branch to guide and reinforce the model’s focus on specific features and regions. To enable automated input prompting, a Rough Mask Generator is employed to generate preliminary glomerular segmentation masks creatively.
- To enhance the segmentation of glomerular boundaries in IF images, we incorporate a hybrid loss function based on DiceLoss, BCELoss, and the novel BoundaryDoULoss in our experiments. BoundaryDoULoss, which designed to focus on edge areas, significantly enhances the accuracy of glomerular boundary recognition.

Through extensive experimental validation, GlomSAM demonstrates significant performance advantages in the critical task of glomerular IF image segmentation for kidney disease diagnosis, achieving a Dice coefficient of 90.15%. This is an improvement of over 15% compared to existing state-of-the-art models.

## Related work

### Glomerular identification and segmentation

Accurate segmentation of glomeruli on WSI is critical for disease diagnosis and treatment planning. Earlier studies of glomerular segmentation relied on image processing techniques and machine learning methods, including Rectangular Histogram of Gradients (R-HOG) [20,21], Segmented Histogram of Gradients (S-HOG), and Support Vector Machines (SVM) [22]. While these methods are effective in processing natural images and simple scenarios, they still have limitations when applied to complex biological tissue images. With the rapid advancement of deep learning technologies, approaches based on deep learning have gradually become the mainstream for glomerular segmentation, significantly enhancing segmentation accuracy and robustness [11,13,23–26]. Hao et al. employed a deep learning-based multimodal framework to classify MN, with the first module designed to segment glomeruli on IF images using the UNet++ segmentation network [24]. Wang et al. introduced Ada-CCFNet, an adaptive weighted confidence-calibrated fusion network, for multimodal direct IF image classification of MN. In the preprocessing stage, a conventional UNet was utilized to segment glomeruli [25]. Yu et al. used a residual UNet architecture integrated with a perceptron and dynamic segmentation head for glomerular segmentation, leveraging mouse renal pathology data to compensate for the scarcity of human renal pathology data [27]. Fu et al. proposed the DeepMT-ND hierarchical task learning framework to enhance the diagnosis of renal disease from low-quality IF images, primarily using ResNet18 for IF image classification [26]. Xia et al. utilized a CNN model trained with the YOLOv5 framework to identify glomeruli in IF images [28].

**Fig 1:**
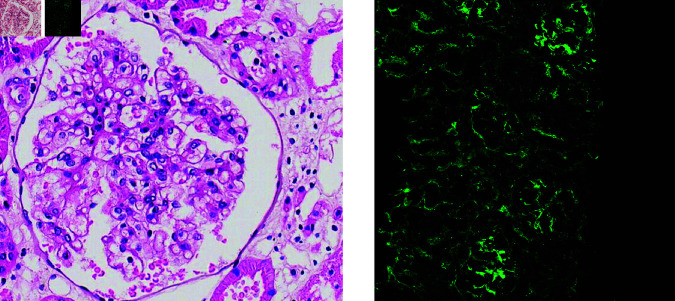
PAS-stained image (left) and IF image (right). The PAS-stained image shows a clear texture, making it easy to see the boundary of glomeruli. However, in the IF image, the texture of the glomeruli appears very similar to the background, which makes it significantly more challenging to distinguish the boundary.

In addition to IF images, H&E and PAS stained images, which exhibit clearer texture structures, are commonly used for glomerular identification due to their relative simplicity compared to fluorescent images (as shown in Fig 1. Lei et al. employed CNN models, including EfficientNet, UNet, and VNet, to perform automatic glomerular identification and classification in conventional PAS stained section images [13]. Feng et al. trained ResNet50 in conjunction with MaskRCNN, Cascade MaskRCNN, DetectoRS, SCNet, QueryInst, and Mask2Former to segment glomeruli in pediatric renal disease pathology images [29]. Gu et al. developed a glomerular segmentation framework based on multi-model integration, combining Full Convolution Networks (FCN), Deeplabv3, and UNet to enhance segmentation robustness and accuracy through an integration strategy [30]. Kaur et al. used a UNet model variant to automatically detect and localize glomeruli in whole-section kidney images [31]. Overall, the previous research mostly used the base model directly without customized strategies.

### Applications of SAM

Various methods have been employed to customize SAM for specific medical images tasks. Hu et al. introduced the SkinSAM model for skin cancer detection by fine-tuning SAM on dermatoscopic images [32]. Instead of training on large-scale natural images, Ma et al. collected extensive medical datasets to train MedSAM that significantly improved SAM’s segmentation performance in the medical field [33]. The success of MedSAM was primarily dependent on large-scale medical data training, without structural modifications of the network. Ding et al. introduced LoRA layers to the image encoder and mask decoder of SAM, using LoRA fine-tuning to create SamLP for license plate detection [34]. Li et al. developed Polyp-SAM for polyp segmentation [35]. Zhang et al. introduced UV-SAM for urban village image segmentation, using a lightweight model called Segformer to provide rough mask input cues for SAM [36]. Wu et al. proposed a method that incorporates domain-specific medical knowledge into the model by adding adapters with a lightweight and effective technique [37].

Glomerular IF images typically exhibit fuzzy boundaries, high noise levels, and low contrast, and other issues. Traditional neural network models, such as UNet-like architectures, often encounter challenges when processing IF images, leading to higher false detection and leakage rates. This poor generalization performance failed to meet the requirements of clinical diagnosis. Besides model limitation, the scarcity of large-scale, high-quality labeled human kidney pathology data further complicates the situation. Maximizing the utility of limited data for training remains a significant challenge in glomerular IF image segmentation. Numerous studies have shown SAM’s stability as a generalized segmentation model across diverse domains and potential on IF images. In this paper, we propose a hybrid customized SAM model to take the processing of pathology images to a higher level by leveraging the strengths of large-scale models and incorporating recent methodological advances.

## Materials and methods

### Data sets preparation

**Fig 2:**
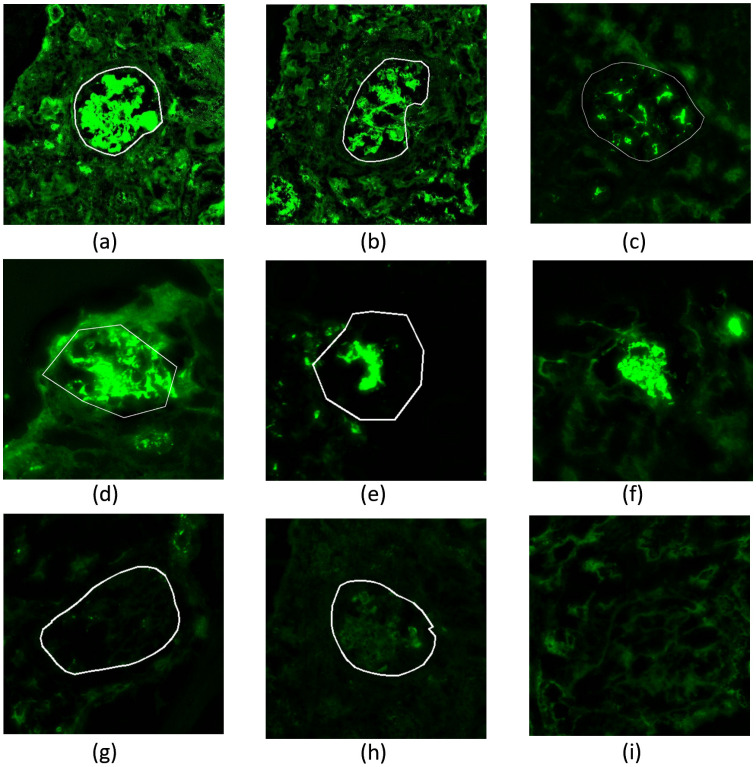
Demonstration of sample diversity. (a-d) Positive lesion glomeruli with various appearances. (e-f) Due to staining contamination or slice location influence, it is difficult to tell if it is a glomeruli. (g-h) Negative glomeruli mixed with background. (i) Background similar to glomeruli.

**Image acquisition:** The images used in this paper are from the retrospective data between January 2022 and September 2023, collected by the cooperative Hangzhou Traditional Chinese Medicine (TCM) Hospital, affiliated with Zhejiang Chinese Medical University. Data were accessed for analysis on September 20, 2023. The authors did not have access to any information that could identify individual participants. All data were anonymized and de-identified before analysis to ensure participant confidentiality. All human kidney tissues were processed for frozen sectioning by standard procedures. Human frozen tissues were sectioned using a freezing microtome at 3 µm. Cryosections were stained with FITC-labeled antibodies of IgA, IgG, IgM, C3, C1q, Kappa, and Lambda. Images were generated using the KF-PRO-400 digital slide scanner (KFbio, Zhejiang Ningbo KonFoong Bioinformation Tech Co. Ltd). All exposure settings were kept the same. To ensure the diversity of experimental data, the IF images included various kinds of glomeruli, such as global sclerosis, glomerular lesions with a positive appearance (coarse granular, fine granular, or linear), distribution (segmental or global), and location (mesangial area, capillary wall, or basement membrane), as shown in Fig 2.

**Fig 3:**
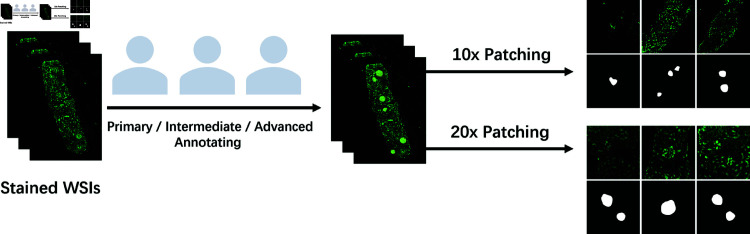
Schematic diagram of the dataset production process. The original WSIs are cropped on magnification 10× and 20× after doctor labeling to obtain the final dataset.

**Manual annotation:** The clinical diagnosis group consisted of three experienced nephropathologists as primary, intermediate, and advanced annotators who independently evaluated the collected images, as shown in Fig 3. The evaluations were conducted in a double-blinded manner, with each pathologist providing assessments of the location and contour of glomeruli, as well as the deposition intensity, morphology, location, and distribution pattern of each glomeruli.

**Construction and preprocess of dataset:** The dataset comprises 933 WSIs from 131 patients, with an average image dimension of almost more than 15,000×15,000 pixels. Through a sliding window, we extracted a total of 10,278 patches of size 1024×1024 pixels from 10× and 20× WSIs. All these images were randomly divided into training set, validation set, and testing set in a ratio of 8:1:1. The final dataset distribution is shown in Table 1.

**Fig 4:**
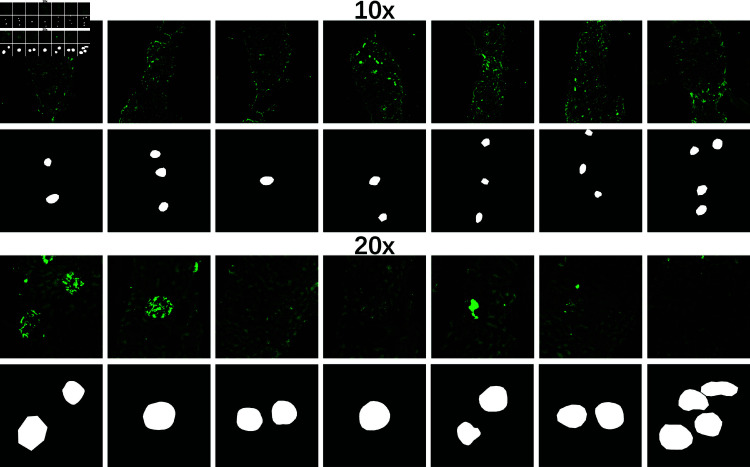
Scale and number of glomeruli in patches from different magnifications.

To comprehensively evaluate the robustness of the model, we did experiments on sub-datasets of different annotators and resolutions, respectively. It can be seen from Fig 4 that the glomeruli in the 10× patches are smaller and harder to recognize, compared to the ones in 20× patches. Most previous studies about glomerular segmentation always have only one glomerulus which occupies most area of the image in cropped patches. In contrast, the dataset utilized in this study contains multiple glomeruli, significantly increasing the segmentation difficulty.

### The overall architecture of GlomSAM

**Fig 5:**
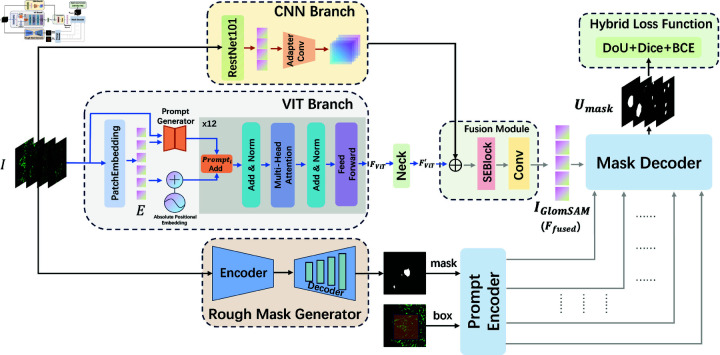
The overall architecture of GlomSAM.

The overall architecture of GlomSAM inherits the image encoder, prompt encoder, and mask decoder from SAM, as shown in Fig 5. The image encoder of GlomSAM integrates ViT and ResNet101 as CNN branch to capture both low-frequency and high-frequency features in the image, leveraging the strengths of both architectures. These features are then fused to generate the final feature information through a feature fusion module. Additionally, a Prompt Generator is employed to enhance the ViT branch’s ability to process glomerular IF image features. Moreover, a Rough Mask Generator is used for automated prompt generation, reducing the burden of manual labeling and improving model accuracy. For the design of the loss function, GlomSAM integrates BinaryCrossEntropyLoss, DiceLoss, and BoundaryDoULoss as a hybrid method to make the model get edge details effectively. The specific improved details of GlomSAM will be described in the following sections.

#### Prompt generator in ViT branch.

**Fig 6:**
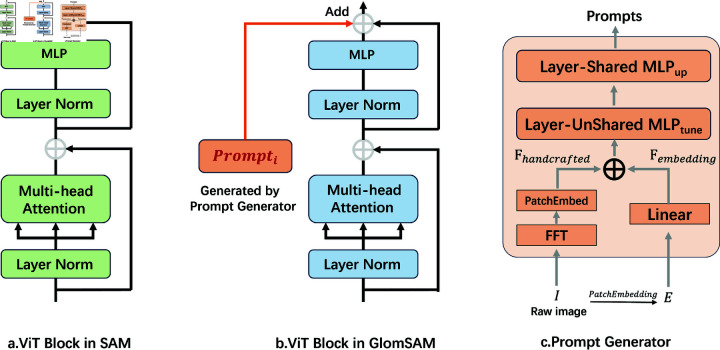
Schematic diagram of prompt generator. a: SAM’s ViT Block. b: Our improved ViT Block. c: Details of the Prompt Generator module.

Fig 6a illustrates SAM’s ViT Block, while Fig 6b depicts our enhanced ViT Block. The Prompt Generator module (shown in Fig 6c) is responsible for generating a series of prompts that serve as input corrections for each layer of the Transformer. These prompts are created by combining handcrafted features $F_{handcrafted}$ and embedding features $F_{embedding}$, aiming to dynamically adjust the attention mechanism of the ViT branch to better adapt to the characteristics of IF images. The features $F_{handcrafted}$ and $F_{embedding}$ are extracted through distinct processes to capture both frequency-domain and spatial-domain information from the input image.

**Table 1:** Distribution of data sets.

Quantity (sheets)	Primary labeling		Intermediate labeling		Advanced labeling	
	10x	20x	10x	20x	10x	20x
**Train**	1112	1452	1246	1646	1317	1766
**Valid**	149	186	170	211	178	223
**Test**	131	175	156	209	170	231

Specifically, the input image *I* undergoes a Fast Fourier Transform (FFT) to extract frequency-domain information. This is followed by a PatchEmbed operation that divides the

frequency-transformed image into patches and embeds them into a lower-dimensional feature space to obtain $F_{handcrafted}$:


$$\begin{array}{ccc} F_{handcrafted}=PatchEmbed(FFT(I)) & & \end{array}$$ (1)

where *PatchEmbed* projects these features into a lower-dimensional representation suitable for processing.

Simultaneously, the image *I* is passed through the PatchEmbedding layer to obtain *E*. A linear transformation then reduces the dimension to produce $F_{embedding}$:


$$\begin{array}{ccc} F_{embedding}=Linear(E) & & \end{array}$$ (2)

The prompt $Prompt_{i}$ for each Transformer layer is generated by combining $F_{handcrafted}$ and $F_{embedding}$, followed by a nonlinear transformation through two lightweight Multi-Layer Perceptrons (MLPs):


$$\begin{array}{ccc} Prompt_{i}=MLP_{up}\left(GELU\left(MLP_{tune}^{i}\left(F_{handcrafted}+F_{embedding}\right)\right)\right),\;i\in [1,depth] & & \end{array}$$ (3)

Here, $MLP_{tune}^{i}$ is the linear layer used to generate task-specific prompts, and $MLP_{up}$ is an up-projection layer with shared weights used to adjust the dimension. *GELU* represents the Gaussian Error Linear Unit activation function.

**Integration and Interaction of Prompts with Transformer Layers**: The generated prompts are seamlessly integrated into each Transformer layer to enhance the feature maps at every stage. Within the ‘ImageEncoderViT‘’s ‘forward‘ method, the ‘PromptGenerator‘ produces a set of prompts corresponding to each Transformer layer. Each $Prompt_{i}$ is reshaped to match the dimensions of the current feature map *x* as (*B*,*H*,*W*,–1), where *B* is the batch size, and *H* and *W* are the height and width of the feature map. During the forward pass, each Transformer block receives its corresponding prompt, which is added to the feature map *x* before being processed by the block. This addition acts as an input correction, injecting both frequency-domain and spatial-domain information into the feature map, thereby guiding the self-attention mechanism to focus more effectively on relevant aspects of the input image. The combination of handcrafted frequency features and learned embedding features ensures that the self-attention mechanism can capture intricate patterns and important regions within the image, facilitating the fusion of multi-scale and multi-level features through distinct lightweight MLP transformations for task-specific adjustments.

**Mechanisms Leading to Performance Enhancement**: Integrating prompts into each Transformer layer leads to several performance improvements. The prompts introduce additional frequency and spatial information, enriching the feature representations processed by the Transformer layers and resulting in more nuanced and discriminative features. By embedding task-specific prompts, the self-attention mechanism is guided to prioritize relevant regions and features within the image, enhancing the effectiveness and accuracy of attention. The dynamic generation of prompts based on input images allows the model to adapt to varying input characteristics, improving its generalization and robustness across diverse datasets. Furthermore, the combination of handcrafted frequency features and learnable embedding features across multiple Transformer layers enables the effective integration of global and local information, leading to superior performance in capturing complex patterns. The use of *GELU* activation and MLP layers introduces nonlinearities that allow the model to capture complex interactions between handcrafted and embedding features, further enhancing the richness of the prompts.

**Necking Layer Processing**: The feature $F_{ViT}^{\prime }$ extracted by the ViT branch is further processed through a necking layer, which consists of convolutional and normalization layers to convert the embedding dimension into the specified output dimension:


$$\begin{array}{ccc} F_{ViT}^{\prime }=Neck(F_{ViT}) & & \end{array}$$ (4)

This necking layer ensures that the ViT features are appropriately scaled and normalized before being fused with the CNN branch features, facilitating effective multi-branch feature integration.

**Final**: By meticulously generating and integrating prompts into each Transformer layer, the Prompt Generator module significantly enhances the feature extraction capabilities of the ViT branch. This integration enriches the feature representations with both frequency and spatial information and dynamically guides the attention mechanism, resulting in improved adaptability and performance of the overall model across diverse input images.

#### The CNN branch and fusion module.

Recent studies [38] have demonstrated that ViT is more focused on low-frequency signals, while CNN is more adept at processing high-frequency signals. In medical image segmentation, many similar studies add CNN branches to SAM [39–41]. In our study, adding the ResNet101, a typical convolutional neural network, enables the model to capture more subtle feature information, thereby improving the segmentation accuracy. The CNN branch we proposed is built on ResNet101, which serves as a powerful backbone for extracting convolutional features from the input image. We removed the final fully-connected and pooling layers, retaining only the convolutional blocks that provide high-level feature maps. Through this step, we preserve the rich spatial information which is crucial for detailed IF image analysis.

The input image *I* is passed through the truncated ResNet101 to produce a feature map consisting of essential local information. We add an Adapter Conv layer to further align these features with the global features extracted by the ViT branch. This layer refines the output feature map from ResNet101 by adjusting its number of channels to match those expected in the later fusion stages. The process above can be expressed as:


$$\begin{array}{ccc} F_{CNN}=AdaptConv(ResNet101(I)),\;F_{CNN}\in {\mathbb{R}}^{B\times C\times H^{\prime }\times W^{\prime }} & & \end{array}$$ (5)

where *B*, *C*, $H^{\prime }$, and $W^{\prime }$ represent the batch size, channel dimension, height, and width of the feature map, respectively. This ensures that the feature $F_{CNN}$ is compatible with subsequent integration.

The next key part is the Fusion Module, which is responsible for combining the convolutional features from ResNet101 with the global context features extracted from the ViT branch. To achieve effective feature fusion, we utilize a fusion module that leverages Squeeze-and-Excitation Blocks (*SEBlock*). The *SEBlock* plays a critical role in recalibrating the feature maps by adaptively assigning importance weights to different channels, allowing the model to focus more on channels that are crucial for IF image segmentation.

Specifically, the *SEBlock* takes $F_{CNN}$ and $F_{ViT}^{\prime }$ as input and combines them to produce a fused representation effectively. This is done by concatenating the features along the channel dimension and then feeding them into the *SEBlock* for channel-wise recalibration:


$$\begin{array}{ccc} F_{fused}=Conv(SEBlock([F_{Vit}^{\prime },F_{CNN}])) & & \end{array}$$ (6)

In short, $F_{fused}$ is the final output of GlomSAM’s image encoder. In our structure, CNN features are adept at recognizing textures and local patterns. They are critical in IF images where subtle differences in tissue can indicate different conditions. ViT features contribute to understanding spatial relationships over the entire image and identifying larger structures. Moreover, the designed fusion strategy enhances the model’s ability to process and analyze complex IF images. By effectively integrating local details and global contextual information, the model achieves a robust representation of the glomerular structures, which is particularly beneficial for challenging cases with weak or ambiguous signal patterns.

#### Rough mask generator.

**Fig 7:**
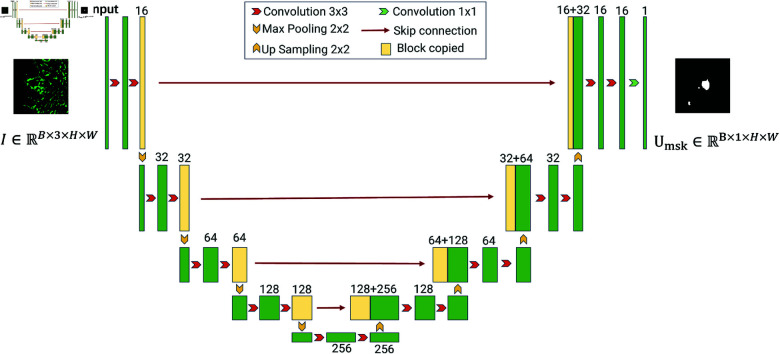
Rough mask generator.

The segmentation performance of the SAM depends on the quality of the input mask prompts. In this section, we introduce the details of Rough Mask Generator as shown in Fig 7.

Rough Mask Generator adopts a typical UNet-style architecture with skip connections for feature fusion. The encoder extracts multi-scale features through repeated convolutions and max pooling operations, progressively reducing the spatial resolution of feature maps. Specifically, the input image passes through two 3x3 convolutions followed by ReLU activation, combined with max pooling to increase feature channels while decreasing spatial dimensions. Skip connections are used to retain features from the encoder for subsequent integration during the decoding process.

The decoder gradually restores the spatial resolution of feature maps through up sampling operations, followed by concatenation of the up-sampled features with corresponding encoder features along the channel dimension. The concatenated features are then refined using two 3x3 convolutions to recover high-resolution details. This upsampling and feature fusion process, repeated at each layer, eventually produces the rough segmentation mask through a 1x1 convolution.

This model architecture effectively generates coarse image masks to enhance the quality of mask prompts by leveraging both local and global feature information. The $U_{mask}$, along with $U_{box}$, which simulates the coarse prompts given by clinicians, is jointly used as inputs to the prompt encoder *Φ* ( *GlomSAM* − *Prompt* )  of GlomSAM. These inputs generate sparse embedded prompts $P_{GlomSAM}$. The process is denoted as:


$$\begin{array}{ccc} P_{GlomSAM}=\Phi_{GlomSAM-Prompt}\left(U_{mask},\;U_{box}\right) & & \end{array}$$ (7)

We use these rough masks as input to the prompt encoder in GlomSAM, guiding the model towards more accurate segmentation results. This iterative process is designed to enhance the quality of the segmentation, with the model learning to focus on areas of interest, thus improving the final output. Although the segmentation mask generated by the Rough Mask Generator is not highly accurate, as a rough cue, its level of accuracy is sufficient.

#### Hybrid loss function.

Existing loss functions for medical image segmentation primarily focus on the overall segmentation accuracy, with limited attention given to guiding the segmentation of boundary regions. Traditional loss functions like Dice Loss and Binary-Cross-Entropy Loss, effective in enhancing overall segmentation quality, often encounter challenges to accurately capture the fine details in boundary regions. To address this limitation, we introduce boundary difference over union Loss (BoundaryDoULoss), proposed by Sun et al., which is specifically designed to optimize boundary segmentation in medical images [42]. The final loss function used for GlomSAM is defined as:


$$\begin{array}{ccc} L=L_{DoU}+L_{Dice}+L_{BCE} & & \end{array}$$ (8)

The design of BoundaryDoULoss is inspired by the Boundary IoU metric, which focuses on the quality of segmented boundaries. This loss function is implemented by calculating the ratio of the difference set to the partial intersection between the predicted and true labels. Specifically, it is defined by the following Eq (9):


$$\begin{array}{ccc} L_{DoU}=\frac{\left(G\cup P)-(G\cap P\right)}{\left(G\cup P)-\alpha \cdot (G\cap P\right)} & & \end{array}$$ (9)

where *G* denotes the true label, *P* denotes the predicted result, and *α* is an adaptive tuning parameter that is automatically adjusted according to the size of the target to focus on the boundary region more reasonably. This enhances BoundaryDoULoss’s ability to handle boundary details, particularly for small or complex glomerular shapes, allowing for more precise edge information capture. *α* is computed as follows:


$$\begin{array}{ccc} \alpha =1-2\times \frac{C}{S},\;\alpha \in [0,1) & & \end{array}$$ (10)

where *C* represents the boundary length of the glomeruli and *S* represents the boundary size of the glomeruli.

In our experiments, we found that directly summing the three loss components provided stable and effective training. The values of these losses are all bounded between 0 and 1, and they focus on different aspects of the task. We found that the model was able to optimize all three aspects simultaneously without the need for introducing weights between the losses.

### Training settings

In terms of training strategies, firstly, the structure of MedSAM is the same as that of SAM, and MedSAM is trained based on SAM by adding medical data sets, having a better performance on IF images. During the training process, we initialize using MedSAM’s weights. Secondly, we first trained the Rough Mask Generator. When training GlomSAM, we froze the parameters of the Rough Mask Generator and utilized the rough masks generated by the Rough Mask Generator by training the prompt encoder in GlomSAM. Although the Rough Mask Generator is not highly accurate, as a rough cue, its level of accuracy is sufficient. Thirdly, we utilized parameter-efficient fine-tuning by freezing the parameters of the image encoder during training. The image encoder has already learned robust image feature representations from large-scale data. Freezing the image encoder allows us to retain these learned features, ensuring that the image encoder does not need to be re-trained. This significantly reduces the number of parameters being updated, making the fine-tuning process much more efficient. By freezing the image encoder, we focus the training on the newly added components, prompt encoder, and mask decoder, which are more specific to our task of glomerular segmentation in kidney pathology.

In experiments, we combined all the training data in a disrupted order for training. All experiments in this study were conducted in a hardware environment with RTX A5000 GPU (with 24 GB of graphics memory), and the model was trained using the Adam optimizer with an initial learning rate set to 1e-4 and a weight decay coefficient of 0.01. To avoid overfitting and ensure the model’s generalization ability, the performance of the validation set is monitored during the training process. Training is halted when the performance stabilizes so that the model does not overfit the training data. To comprehensively evaluate the model performance, we adopt the following four main evaluation metrics: Pixel Accuracy, IOU (Jaccard), Dice Score, and Recall.

### Ethics statement

The authors confirm that all methods were carried out in accordance with relevant guidelines and regulations. This study and all experimental protocols were approved by the Ethics Committee of the Hangzhou Traditional Chinese Medicine (TCM) Hospital (2023LL018). The need for informed consent was waived by the ethics committee.

## Results

To show the performance of GlomSAM, we conducted comparison experiments with state-of-the-art models which were often used by other glomerular researches such as UNet [43], UNet++ [44], SwinUNet [45], TransUNet [46], SegNet [47], Yolov8 [48], Yolact [49], MaskRCNN [50], SAM [17], MedSAM [33], and so on. In this section, we first quantitatively compare the different models to see the performance through the metrics; Secondly, we qualitatively analyze the segmentation results of the different models; And lastly, we conducted ablation experiments on GlomSAM to compare the impact and importance of the different components.

### Quantitative comparison of different models

Due to the differing observation results among physicians, we created box prompts based on the labeling information of different physicians to simulate the experience of different physicians during the experimental tests. To measure the stability and robustness of the models, all models in the quantitative analysis were tested with different levels of labeling on test sets of 10× images. To further investigate the impact of image magnification on model performance, we also conducted tests on 20× images.

Table 2 illustrates the average values of key test indicators for each model on the 10× images, providing a comparative overview of model performance. GlomSAM consistently outperformed all other models across all expertise levels. For primary labeling, GlomSAM achieved a Dice of 84.45%, surpassing MedSAM’s 81.76%, and SAM’s 78.01%. The IoU for GlomSAM was 73.15%, and the Recall was 84.97%. In contrast, UNet and UNet++ recorded significantly lower Dice of 59.58% and 58.37%, respectively, highlighting their limitations in capturing complex features on this magnification. SwinUNet and TransUNet demonstrated suboptimal performance with Dice of 35.94% and 49.89%, respectively. Yolov8 achieved a higher Recall of 80.08% but had a moderate Dice of 65.72%, indicating a trade-off between detection ability and segmentation accuracy. Yolact showed similar trends with a Dice of 58.04% and a Recall of 57.04%. SegNet performed reasonably well among traditional models with a Dice of 64.21%. Similar trends were observed for Intermediate and Advanced labeling, with GlomSAM maintaining top performance.

**Table 2:** Test results of different models on 10× images (Rounded to two decimal places).

Models	Primary Labeling				Intermediate Labeling				Advanced Labeling			
	Dice	IoU	Accuracy	Recall	Dice	IoU	Accuracy	Recall	Dice	IoU	Accuracy	Recall
UNet [43]	59.58	46.28	97.89	69.75	61.95	49.16	98.01	68.27	60.39	47.72	97.90	64.45
UNet++ [44]	58.37	45.45	97.73	72.57	63.12	50.20	98.00	74.61	62.92	50.20	97.98	71.24
SwinUNet [45]	35.94	26.80	97.08	33.98	36.33	27.00	96.94	32.41	31.65	22.99	96.65	27.82
TransUNet [46]	49.89	38.98	97.90	46.67	49.57	38.09	97.80	46.43	46.64	35.56	97.65	42.24
SegNet [47]	64.21	51.14	97.94	72.84	68.37	55.47	98.12	75.09	68.27	55.46	98.09	72.30
Yolov8 [48]	65.72	52.74	95.87	80.08	68.10	55.73	95.78	79.41	69.61	57.24	95.69	78.91
Yolact [49]	58.04	46.31	98.23	57.04	60.43	48.90	98.28	56.50	59.23	47.48	98.15	54.16
MaskRCNN [50]	34.89	23.55	95.61	82.24	35.31	23.55	95.40	82.50	36.21	24.10	95.51	81.00
SAM [17]	78.01	64.78	98.74	76.59	77.54	64.15	98.73	75.75	75.45	61.16	98.50	72.76
MedSAM [33]	81.76	70.22	99.02	78.74	82.57	70.82	99.01	79.61	80.31	67.76	98.87	75.98
**GlomSAM (Ours)**	**84.45**	**73.15**	**99.06**	**84.97**	**85.17**	**74.82**	**99.08**	**85.13**	**82.97**	**72.50**	**98.92**	**82.96**

**Fig 8:**
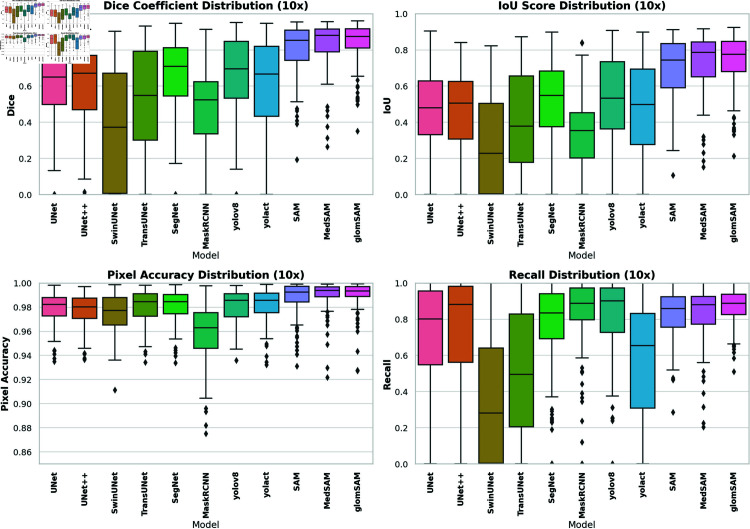
Distribution of model metrics on 10× images. From the distribution graph, the segmentation effect of each model can be compared intuitively. It can be seen that the segmentation performance of the traditional models is unstable while the SAM series models have excellent stability.

Fig 8 details the distribution of these indicators, offering deeper insights into their variability and distribution patterns across different models. The box plot shows a broad range of Dice scores for traditional models like UNet and UNet++, with numerous outliers indicating unstable performance. In contrast, the SAM series models (SAM, MedSAM, GlomSAM) display more concentrated distributions, reflecting higher and more consistent Dice scores. Particularly, our proposed GlomSAM has a higher median and narrower interquartile range, indicating its consistent and reliable segmentation performance across different tasks.

Table 3 provides a comparative overview of model performance on the 20× images. All models showed improved performance due to higher resolution. GlomSAM achieved a Dice of 89.50% and an IoU of 81.58% for Primary labeling, surpassing MedSAM’s 87.54% and SAM’s 83.93%. The Recall increased to 89.60%, indicating enhanced sensitivity to finer details. UNet and UNet++ showed moderate gains but remained behind, with Dice of 61.81% and 63.59%, respectively. SwinUNet and TransUNet improved slightly to Dice of 61.67% and 66.08%, yet still lagged behind the SAM series models. Yolov8 and Yolact exhibited higher Recall rates (84.74% and 81.39%) but had lower Dice (72.27% and 70.70%). SegNet performed relatively well among traditional models with a Dice of 71.01%. Similar trends are observed at Intermediate and Advanced labeling levels. GlomSAM consistently outperformed other models, achieving Dice of 90.15% (IoU 82.47%) for Intermediate labeling and 89.89% (IoU 82.06%) for Advanced labeling.

**Table 3:** Test results of different models on 20× images (rounded to two decimal places).

Model	Primary Labeling				Intermediate Labeling				Advanced Labeling			
	Dice	IoU	Accuracy	Recall	Dice	IoU	Accuracy	Recall	Dice	IoU	Accuracy	Recall
UNet [43]	61.81	49.83	95.53	63.61	64.04	51.80	95.75	65.03	62.47	50.19	95.54	61.75
UNet++ [44]	63.59	52.04	95.67	65.34	65.10	53.13	95.82	66.06	63.54	51.34	95.65	62.70
SwinUNet [45]	61.67	49.95	94.24	65.96	61.89	50.35	94.22	65.54	58.61	47.56	93.78	61.77
TransUNet [46]	66.08	53.08	94.62	70.53	66.68	53.46	94.68	70.73	64.17	50.86	94.33	67.37
SegNet [47]	71.01	59.96	96.25	70.97	73.82	62.78	96.47	73.30	71.40	60.10	96.23	68.55
Yolov8 [48]	72.27	60.51	89.23	84.74	75.52	63.49	89.39	86.90	75.63	63.47	89.38	85.63
Yolact [49]	70.70	58.24	95.14	81.39	73.37	60.94	95.63	83.17	73.02	60.32	95.48	82.05
MaskRCNN [50]	61.10	49.19	95.04	68.31	62.87	50.82	95.32	68.84	60.85	48.69	95.14	66.08
SAM [17]	83.93	73.06	97.64	79.66	85.10	74.53	97.84	80.57	84.72	74.20	97.66	79.33
MedSAM [33]	87.54	78.63	98.18	84.91	88.63	79.83	98.33	85.66	88.59	79.90	98.22	84.69
**GlomSAM (Ours)**	**89.50**	**81.58**	**98.35**	**89.60**	**90.15**	**82.47**	**98.45**	**91.01**	**89.89**	**82.06**	**98.37**	**90.64**

**Fig 9:**
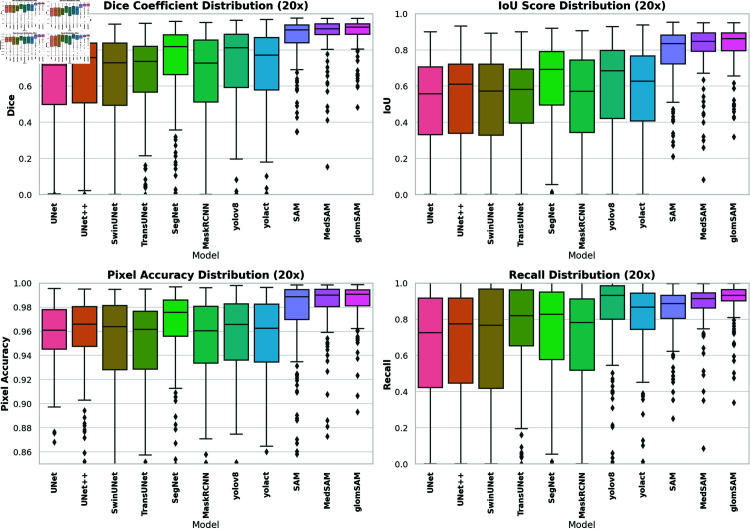
Distribution of model metrics on 20× image. It shows evident improvement in the performance of all models compared to 10× images, and most of the models have stable performance.

The box plots further illustrate the distribution of key metrics (Dice, IoU, accuracy, and recall) on 20× images in Fig 9. Compared to the 10× results, most models show narrower interquartile ranges, indicating improved stability. The SAM series models, particularly GlomSAM, maintain superior performance with minimal variability, while models like UNet and SwinUNet exhibit more pronounced fluctuations, suggesting less consistency in their segmentation outputs.

### Qualitative comparison of different models

This section distinguishes between different magnifications. We first compared GlomSAM with SAM and MedSAM, and then compared GlomSAM with other state-of-the-art models.

**Fig 10:**
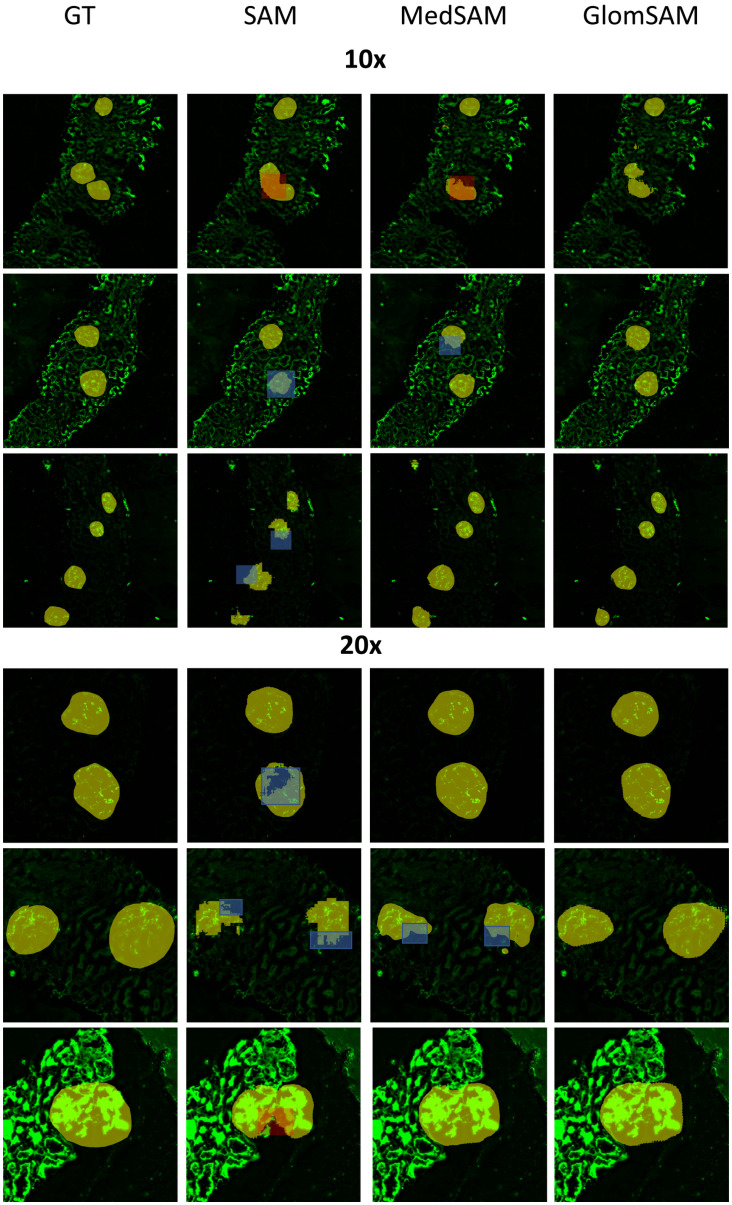
Sample diagram of the SAM series models. Here, we mark the raw image with different color areas. The yellow area in the image indicates the ground truth in the first column and the segmentation results of models in other columns. The red box marker indicates that the model cannot segment the accurate boundary. The blue box marker cannot segment the boundary in a detailed and rounded way with a jagged shape.

As shown in Fig 10, the red box markers of SAM and MedSAM models were unable to segment the accurate boundary while GlomSAM had an obvious boundary. The blue box markers of SAM and MedSAM models did not segment the boundary in a detailed and rounded way with a jagged shape. In such cases, SAM and MedSAM failed to accurately segment glomeruli. Through our proposed modifications, GlomSAM indicates significant improvements in this situation.

**Fig 11:**
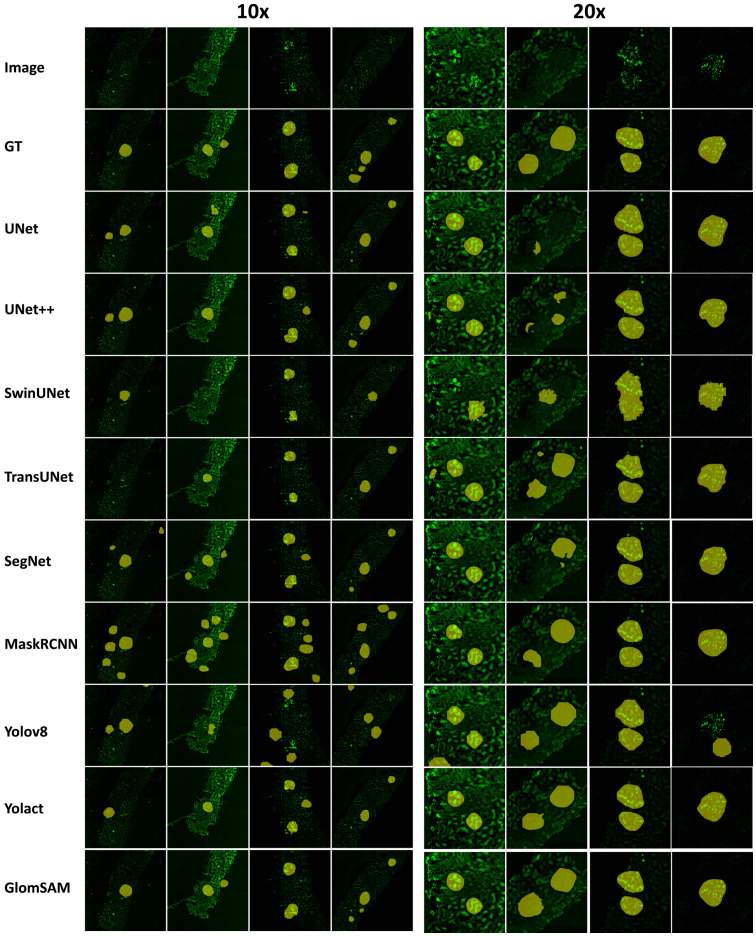
Qualitative comparison results between GlomSAM and other models.

Fig 11 demonstrates the segmentation results of GlomSAM and other state-of-the-art models. On 10× images, while multiple models such as MaskRCNN could find the glomeruli within their segmented regions, they also encompassed many backgrounds. Besides, SwinUNet and TransUNet frequently failed to segment the glomeruli at all, leading to poor outcomes. In contrast, Yolov8, Yolact, SegNet, and GlomSAM were able to correctly segment most of the glomerular regions, showing superior performance. On 20× images, the segmentation results improved significantly across all models. Notably, GlomSAM got the best robustness and stability, with relatively consistent performance on both two magnifications.

### Ablation study

To evaluate the contribution of each component to the model performance, we designed and conducted a series of ablation experiments. The experiments mainly include four key components: BoundaryDoULoss(DoULoss), Rough Mask Generator, Prompt Generator and CNN branch. We used the dataset with advanced labels in the ablation study and MedSAM as the base model without adding any of our proposed improvements.

Our ablation study(As shown in Table 4) reveals that DouLoss contributes the most significant impact, with its removal causing substantial performance degradation in IoU scores (decreasing by 2.46% at 10x and 2.16% at 20x). Importantly, the four components work synergistically. Their combined implementation achieves optimal performance across all metrics (Dice: 89.89%, IoU: 82.06%, Recall: 90.64% at 20x), consistently outperforming versions with any single component removed.

**Table 4:** Ablation study results.

Components				10×			20×		
DoULoss	Rough Mask	Prompt	CNN	Dice	IoU	Recall	Dice	IoU	Recall
	Generator	Generator	Branch						
×	×	×	×	80.31	67.76	75.98	88.59	79.90	84.69
×	✓	✓	✓	81.65	70.04	82.89	89.10	80.92	89.79
✓	×	✓	✓	83.10	71.77	80.04	89.27	81.05	87.71
✓	✓	×	✓	83.29	72.20	82.50	89.30	81.19	88.47
✓	✓	✓	×	**83.23**	72.22	81.67	89.41	81.35	89.62
✓	✓	✓	✓	82.97	**72.50**	**82.96**	**89.89**	**82.06**	**90.64**

When testing on both 10x and 20x datasets, removing any single component generally leads to performance degradation. While removing certain components occasionally yields slightly higher Dice scores in the 10x scenario, the complete model achieves the most balanced and optimal performance across all metrics. The comprehensive evaluation validates that these components work synergistically to enhance the model’s overall performance, with each component contributing to the system’s robustness and effectiveness.

## Discussion and conclusion

CKD seriously endangers human health and the social economy globally. IF images are the gold standard for diagnosing some kinds of kidney disease such as IgAN and MN. Glomerular detection and segmentation in IF images are the first crucial step of automated assisted diagnosis. In this paper, we propose the GlomSAM, which is based on SAM and customized for glomerular detection and segmentation in IF images. In GlomSAM, a CNN branch and Prompt Generator are added to strengthen the model’s ability. Furthermore, we employed a Rough Mask Generator to automatically generate preliminary mask and design a hybrid loss function to improve glomerular boundary segmentation.

One key advantage of our research is our analysis of several critical factors of segmentation. In particular, our efforts strive to understand what works best for IF images. By studying the effort of resolution and labels through different levels of experts, we are better able to understand how to improve the current pipeline that operates on IF images to yield better results. For the effect of the resolution, both Dice and Recall are much better on 20× images through the comparison between 10× and 20× magnification in depth. For the effect of different level labels, the performance gap narrows as the resolution grows, suggesting that our model can tolerate the small labeling error. Another key advantage is how our study definitively shows that our proposed framework can yield a clear advantage in accuracy and recall over the other state-of-art models which were widely used by other reference researches.

However, there are some important limitations to our study. First, the focus of this study was in the context of renal pathology and glomerular data on IF images. However, we expect the findings will be generalizable for other fields on IF images as the scaling issues are similar. Another limitation includes the fundamental restraints of the GPU when processing large-scale images. In the future, it will be beneficial to experiment on the WSI rather than the cropped parches. Furthermore, integrating the model into clinical workflows and obtaining feedback from medical professionals will help refine and validate its practical utility.

In conclusion, we propose GlomSAM, the first semantic segmentation algorithm based on SAM for glomerular processing on IF images, achieving a high-performance segmentation model with a limited amount of training data. This result demonstrates the powerful potential of GlomSAM in the field of glomerular fluorescence image processing and lays the foundation for further clinical applications.
